# Increased hepatotoxicity among HIV-infected adults co-infected with *Schistosoma mansoni* in Tanzania: A cross-sectional study

**DOI:** 10.1371/journal.pntd.0005867

**Published:** 2017-08-17

**Authors:** Amon I. Marti, Soledad Colombe, Peter J. Masikini, Samuel E. Kalluvya, Luke R. Smart, Bahati M. Wajanga, Hyasinta Jaka, Robert N. Peck, Jennifer A. Downs

**Affiliations:** 1 Catholic University of Health and Allied Sciences and Bugando Medical Centre, Mwanza, Tanzania; 2 Center for Global Health, Department of Medicine, Weill Cornell Medicine, New York, New York, United States of America; Brown University, UNITED STATES

## Abstract

**Introduction:**

Little is known about hepatotoxicity in patients with schistosome and HIV co-infections. Several studies have reported increased liver enzymes and bilirubin levels associated with schistosome infection. We investigated whether HIV-infected adults on antiretroviral therapy who had *S*. *mansoni* co-infection had a higher prevalence of hepatotoxicity than those without.

**Methodology/Principal findings:**

We determined the presence and grade of hepatotoxicity among 305 HIV-infected outpatients who had been on medium-term (3–6 months) and long-term (>36 months) antiretroviral therapy in a region of northwest Tanzania where *S*. *mansoni* is hyperendemic. We used the AIDS Clinical Trial Group definition to define mild to moderate hepatotoxicity as alanine aminotransferase, alanine aminotransferase, and/or bilirubin elevations of grade 1 or 2, and severe hepatotoxicity as any elevation of grade 3 or 4. We determined schistosome infection status using the serum circulating cathodic antigen rapid test and used logistic regression to determine factors associated with hepatotoxicity. The prevalence of mild-moderate and severe hepatotoxicity was 29.6% (45/152) and 2.0% (3/152) in patients on medium-term antiretroviral therapy and 19.6% (30/153) and 3.3% (5/153) in the patients on long-term antiretroviral therapy. *S*. *mansoni* infection was significantly associated with hepatotoxicity on univariable analysis and after controlling for other factors associated with hepatotoxicity including hepatitis B or C and anti-tuberculosis medication use (adjusted odds ratio = 3.0 [1.6–5.8], p = 0.001).

**Conclusions/Significance:**

Our work demonstrates a strong association between *S*. *mansoni* infection and hepatotoxicity among HIV-infected patients on antiretroviral therapy. Our study highlights the importance of schistosome screening and treatment for patients starting antiretroviral therapy in schistosome-endemic settings. Additional studies to determine the effects of schistosome-HIV co-infections are warranted.

## Author summary

*Schistosoma sp*. are parasitic worms that infect at least 218 million people worldwide. Over 90% of these individuals live in Africa, where HIV infection is also endemic. Schistosome worms lay eggs that damage the gastrointestinal and genitourinary tracts, causing extensive morbidity and mortality. Patients who have HIV and *Schistosoma mansoni* co-infections are at risk for damage to the liver due to both the effects of the schistosome parasite and the side-effects of antiretroviral therapy. However, little is known about the additional liver effects of schistosome infection in patients already taking antiretroviral therapy. Therefore, we conducted a study in northwest Tanzania, where our prior work has shown that approximately one-third of HIV-infected patients also have schistosome infections, to investigate the effect of co-infection with *Schistosoma mansoni* on liver damage in patients taking antiretroviral therapy. We studied 305 HIV-infected outpatients on medium and long-term antiretroviral therapy and determined both liver damage and *S*.*mansoni* infection in those patients. We found that among patients on antiretroviral therapy, those with HIV-schistosome co-infection were 3 times more likely to have liver damage than those with HIV infection alone. Our work shows the importance of screening and treating for *Schistosoma mansoni* to decrease the risk of liver damage in patients infected with HIV.

## Introduction

Hepatotoxicity increases mortality and morbidity among HIV-infected patients on antiretroviral therapy (ART), especially those with high CD4^+^ T-lymphocyte (CD4) counts at initiation of ART [1]. The prevalence of hepatotoxicity due to ART is expected to increase in sub-Saharan Africa as ART becomes more widely available and as patients initiate treatment at higher CD4 counts [2]. Most studies on hepatotoxicity in HIV-infected patients on ART have been conducted in the first 3 months of ART, when the majority of hepatotoxic reactions are believed to occur and when many patients are also receiving anti-tuberculosis treatment [3]. Only a few studies have assessed hepatotoxicity in HIV-infected patients on ART for more than 3 months. Two small studies have shown that both hepatitis B and hepatitis C are associated with hepatotoxicity in HIV-infected patients on ART for more than one year [4–5]. Beyond this, little is known about hepatotoxicity in HIV-infected patients on long-term ART.

*Schistosoma* sp. are parasitic worms that infect at least 218 million people worldwide [6]. *Schistosoma mansoni* alone infects more than 83 million people, primarily in Africa, South America and the Caribbean [7]. In regions of Tanzania in which *S*. *mansoni* is highly endemic, an estimated 30–50% of HIV-infected patients have *S*. *mansoni* co-infection [8]. Classic teaching on *S*. *mansoni* is that its eggs cause Symmer’s pipestem fibrosis with resultant portal hypertension, while preserving hepatocellular function [9–10]. However, several studies have reported that schistosome infection may additionally lead to elevated liver enzymes and bilirubin levels [11–13]. To our knowledge, no studies have investigated the effects of *S*. *mansoni* infection on liver function in HIV-infected patients on ART.

Therefore, our objectives were: (1) to assess the prevalence of hepatotoxicity in HIV patients on medium-term and long-term ART and (2) to determine whether HIV-infected adults on ART who had *S*. *mansoni* co-infection had a higher prevalence of hepatotoxicity than HIV-infected adults on ART without schistosome co-infection.

## Methods

### Study site and study design

Bugando Medical Centre (BMC) is a referral hospital located in Mwanza city, on the southern shore of Lake Victoria, Tanzania. In this region, the prevalence of HIV is 5% [14]. We conducted a cross-sectional study in outpatients seeking care at BMC’s HIV clinic, which has registered approximately 15,000 patients since its opening in 2004. On average, 45 patients per month are started on ART. Our team's clinical experience working at the BMC HIV clinic over the past five years demonstrates that approximately 1/3 of HIV-infected patients have *S*. *mansoni* infection, and < 1% have *S*. *haematobium*.

### Study participants

To assess for hepatotoxicity in patients using ART in the medium and longer term, we focused on two groups: patients who had been on ART for 3–6 months (GROUP 1), and patients who had been on ART for >36 months (GROUP 2). We calculated that enrolling 152 adults in each group would provide 80% power to detect a difference in hepatotoxicity among those with versus without schistosome infection. Calculations were based on the prediction that 5% of HIV-infected adults without schistosome infection would have hepatotoxicity, compared to 20% of those with HIV-schistosome co-infection [13].

All adults >18 years old who were attending BMC HIV clinic between August and December 2014, who provided written informed consent and had been on ART for 3–6 months or >36 months were eligible for enrolment. We excluded patients with known poor adherence to ART, defined according to the Tanzanian national HIV Care and Treatment Program as missing more than 3 doses or more than 2 days of ART, as assessed monthly by nurses and physicians at the HIV clinic. Assessments were made both by patient report and by pill counts.

### Data collection and laboratory testing

Patients were recruited consecutively until the predetermined sample size was reached. Baseline information for each patient was obtained from the HIV clinic database and patients’ files. We interviewed patients to obtain history and demographic information, and performed physical examinations that included weight, height, and liver span measurements. Liver ultrasound was performed in all patients to determine the portal vein diameter. Urine was tested for *Schistosoma mansoni* using a point-of-care urine Circulating Cathodic Antigen dipstick test (Rapid Medical Diagnostics, Pretoria, South Africa). Results of the CCA test were recorded as negative, 1 for a faintly visible test line, 2 for a test line that was equal in intensity to the control line, and 3 for a test line that was higher intensity than the control line, according to the manufacturer’s statement that the intensity of the test line is qualitatively related to the intensity of the schistosome infection. All participants provided serum and plasma samples to the BMC clinical laboratory for determination of Alanine Aminotransferase (ALT), Aspartate Aminotransferase (AST) bilirubin levels, CD4 counts, and the presence of hepatitis B surface antigens and hepatitis C antibodies.

Liver enzymes and bilirubin levels were quantitated using the COBAS Integra 400 Plus machine (Roche, Basel, Switzerland). Hepatitis B was tested by surface Antigen rapid test (Laborex IVD Italiano SRL, Milan, Italy) and hepatitis C by antibody rapid test (Guangzhou Wondfo Biotech Co, Ltd, Guangzhou, China). CD4 levels were determined using the BD Tritest CD3/CD4/CD45 (BD Biosciences, San Jose, California, USA).

### Statistical analysis

Analysis was done using Microsoft Excel and Stata/IC version 13 (College Station, Texas). We used the AIDS Clinical Trial Group definition of hepatotoxicity to define mild to moderate hepatotoxicity as AST, ALT, and/or bilirubin elevations of grade 1 or 2, and severe hepatotoxicity as AST, ALT, and/or bilirubin elevation of grade 3 or 4. For AST and ALT these groups are 1.25–5 and >5 times the upper limit of normal, and for bilirubin these groups are 1.1–2.9 and >2.9 times the upper limit of normal. Categorical variables were described as proportions and were compared between GROUP 1 and GROUP 2 using the chi-squared or Fisher’s Exact test while continuous variables were summarized by medians and interquartile ranges and compared using the Wilcoxon rank-sum test.

When an outcome had a value of less than 5 on the chi-squared analysis, we determined the strength of the association using Fisher’s Exact test. All variables that showed an association (p<0.10) with any hepatotoxicity on univariable analysis were subjected to Firth multivariable logistic regression analysis to identify the factors independently associated with hepatotoxicity among HIV-infected adults using ART due to the presence of several factors that were significant and had a small number of outcomes [15].

### Ethics

Ethical approval to conduct this study was obtained from the Joint Research and Publication Committee of the Catholic University of Health and Allied Sciences and BMC (CREC/067/2015). All results were communicated to patients’ primary care physicians. Patients found to have hepatitis B co-infection were given tenofovir-based regimens and those with *S*. *mansoni* were given praziquantel.

## Results

At the time of the study, the clinic had 3,971 patients receiving ART. Of these, 2,486 had been taking ART for 3–6 months or for more than 36 months, of which 17 were noted to have poor adherence to medications and were excluded. We approached 309 consecutive patients who were being seen at the clinic during the study period. Of these, four were not willing to participate and the remaining 305 were enrolled in the study (152 in GROUP 1 and 153 in GROUP 2). Patients in GROUP 1 had been on ART for a median of 4.4 [3.6–5.4] months, and those in GROUP 2 had been on ART for 76.4 [51.2–91.3] months.

There were significantly more females in GROUP 2 than in GROUP 1 (75.8% (116/153) versus 62.5% (95/152), p = 0.01). GROUP 2 patients were also significantly older (41 [37–47] versus 38 [34–48] years, p = 0.001). Significantly fewer patients in GROUP 2 reported their occupation as “peasant/farmer” compared to GROUP 1 (14/153, 9.2% versus 31/152, 20.4%, p = 0.006). There was a significantly higher use of anti-TB medication in GROUP 1 (11.2% (17/152) versus 3.3% (5/153), p = 0.008, Table 1).

**Table 1:** Baseline characteristics of 305 HIV-infected, Tanzanian adults on antiretroviral therapy (ART) at Bugando Medical Centre and comparison between patients on ART 3–6 months (GROUP 1) or >36 months (GROUP 2).

	GROUP 1 (N = 152): Number(%) or Median [IQR]	GROUP 2 (N = 153): Number (%) or Median [IQR]	p-value
**Time on ART (months)**	4.4 [3.6–5.4]	76.4 [51.2–91.3]	<0.0001
**Gender**			
Male	57 (37.5)	37 (24.2)	0.013
Female	95 (62.5)	116 (75.8)	
**Age in years**	38 [34–48]	41 [37–47]	0.001
**Residence**			
Urban	103 (67.8)	122 (79.7)	0.019
Rural	49 (32.2)	31 (20.3)	
**Occupation**			
Farmer	31 (20.4)	14 (9.2)	0.006
Petty trader	59 (38.8)	75 (49.0)	0.073
**Current ART regimen**			
AZT/3TC/EFV	36 (23.7)	33 (21.5)	< 0.001
TDF/3TC/EFV	88 (57.9)	21 (13.7)	
AZT/3TC/NVP	0 (0.0)	47 (30.7)	
TDF/FTC/EFV	28 (18.4)	48 (31.4)	
ABC/3TC/LPV/r	0 (0.0)	4 (2.6)	
**Change in ART during treatment**	0 (0.0)	76 (49.7)	< 0.001
**Current use of anti TB medications**	17 (11.2)	5 (3.3)	0.008
**Using local herbs medication**	10 (6.6)	17 (11.1)	< 0.001
**Body mass index (kg/m**^**2**^**)**	21.4 [19.5–23.7]	22.3 [18.4–25.9]	< 0.001

Mild-moderate hepatotoxicity affected 29.6% (45/152) of adults in GROUP 1 and 19.6% (30/153) in GROUP 2 (p = 0.04, Table 2). Three patients in GROUP 1 and five patients in GROUP 2 had severe hepatotoxicity. Two of these, both in GROUP 2, were using nevirapine, and the other six were on efavirenz-based regimens.

**Table 2:** Laboratory findings among 305 HIV-infected Tanzanian outpatients on antiretroviral therapy.

Finding	HIV-infected adults on ART for 3–6 months Number (%) (N = 152)	HIV-infected adults on ART for >3 years Number (%) (N = 153)	p-value for difference
**AST U/L**			
Grade 0 (≤ 50)	110 (72.4)	123 (80.4)	0.056
Grade 1 (> 50 and ≤ 100)	32 (21.1)	16 (10.5)	
Grade 2 (> 100 and ≤ 200)	7 (4.6)	9 (5.9)	
Grade 3 (> 200 and ≤ 400)	2 (1.3)	5 (3.3)	
Grade 4 (> 400)	1 (0.7)	0	
**ALT U/L**			
Grade 0 (≤ 51.3)	122 (80.3)	142 (92.8)	0.006
Grade 1 (≤ 102.5)	24 (15.8)	9 (5.9)	
Grade 2 (≤ 205)	4 (2.6)	1 (0.7)	
Grade 3 (≤ 410)	2 (1.3)	1 (0.7)	
Grade 4 (> 410)	0	0	
**Total bilirubin ul/L**			
Grade 0 (≤ 18.7)	147 (96.7)	151 (98.7)	0.29
Grade 1 (≤ 25.5)	2 (1.3)	2 (1.3)	
Grade 2 (≤ 49.3)	3 (2.0)	0	
Grade 3 (≤ 85)	0	0	
Grade 4 (> 85)	0	0	
**Mild-moderate hepatotoxicity (Grade 1–2) based on any parameter**	44 (28.9)	29 (19.0)	0.043
**Severe hepatotoxicity (Grade 3–4) based on any parameter**	3 (2.0)	5 (3.3)	0.37
**Any hepatotoxicity**	47 (30.9)	34 (22.2)	0.085
**Hepatitis B antigen positivity**	8 (5.3)	5 (3.3)	0.39
**Hepatitis C antibody positivity**	2 (1.3)	3 (2.0)	1.0
***S*. *mansoni* CCA test result**			
0	109 (71.7)	136 (88.9)	<0.001
1	29 (19.1)	8 (5.2)	
2	14 (9.2)	9 (5.9)	
3	0	0	

In total, 8 patients had severe hepatotoxicity. Among these patients, 1 had both hepatitis B and C, 2 were using anti-tuberculosis medications and were also schistosome antigen positive, 2 had hepatitis B alone, and 1 reported herbal medication use. The remaining 2 patients with severe hepatotoxicity were negative for *S*. *mansoni*, hepatitis B, hepatitis C, alcohol use, and anti-tuberculosis medications. These two patients had both been on ART for 3–6 months, and both reported a history of lake water contact and rice cultivation.

Factors associated with hepatotoxicity are presented in Table 3. On multivariable analysis by Firth logistic regression, factors that remained significantly associated with hepatotoxicity included: hepatitis B surface antigen positivity (OR = 122 [7–2121], p = 0.001), use of anti-tuberculosis medication (OR = 42 [2–803], p = 0.014), hepatitis C antibody positivity, (OR = 6.2 [2.5–15.8], p<0.001) and schistosome antigen positivity (OR = 3.0 [1.6–5.8], p = 0.001). In addition to the factors listed in Table 3, contact with lake water (OR = 1.9 [1.1–3.2], p = 0.013) and history of cultivating rice (OR = 2.3 [1.3–3.8], p = 0.002) were also associated with hepatotoxicity by univariable analysis.

**Table 3:** Factors associated with hepatotoxicity in patients on ART in Tanzania.

Characteristic	Hepatotoxicity N(%) or median (IQR) (n = 81)	No hepatotoxicity N(%) or median (IQR) (n = 224)	Univariable Analysis–Odds Ratio [95%CI]	p-value	Multivariable Analysis–Odds Ratio [95%CI]	p-value
**Age (years)**	40 [33–45]	39 [32–45]	1.00 [0.98–1.03]	0.67		
**Female gender**	55 (67.9)	156 (69.6)	0.9 [0.5–1.6]	0.77		
**BMI (kg/m**^**2**^**)**	21.5 [18.7–24.2]	21.9 [20.0–24.6]	0.96 [0.90–1.02]	0.19		
**Current CD4 count (cells/μL)**	272 [183–390]	319 [202–535]	0.999 [0.998–1.000]	0.17		
**Use of herbs**	10 (12.4)	17 (7.6)	1.7 [0.8–3.9]	0.20		
**Alcohol consumption**	20 (24.7)	43 (19.2)	1.4 [0.8–2.5]	0.30		
**ART duration (months)**	5.5 [3.9–62.3]	38.3 [4.4–77.0]	0.99 [0.99–1.0]	0.13		
Hepatitis B*	13 (16.1)	0	---**	**<0.001**	122 [7–2121]	**0.001**
Hepatitis C*	5 (6.2)	0	---**	**0.001**	6.2 [2.5–15.8]	**<0.001**
*S*. *mansoni* antigen positive*	27 (33.3)	33 (14.7)	2.9 [1.6–5.2]	**<0.001**	3.0 [1.6–5.8]	**0.001**
Anti-TB drug use*	14 (17.3)	8 (3.6)	5.6 [2.3–14.0]	**<0.001**	42 [2–803]	**p = 0.014**

*Variables that remained significantly associated with hepatotoxicity on multivariable analysis.

**Odds ratio not calculated due to small numbers; Fisher’s Exact test used instead.

In addition, *S*. *mansoni* infection was associated with higher transaminases: ALT in those with *S*. *mansoni* was 32 [19–57] U/L versus 26 [16–37] in those without, and AST was 39 [24–68] versus 35 [25–47] (p = 0.018 and p = 0.088, respectively). Portal vein diameter was also larger in those with schistosome infection: 11.0 [10.0–12.3] versus 10.4 [9.8–11.2] cm (p = 0.0098). Liver span was larger as well (10 [9–11] versus 9 [9–10] cm, p = 0.0024).

The association of *S*. *mansoni* infection with hepatotoxicity was also assessed separately in the two groups. *S*. *mansoni* infection was significantly associated with hepatotoxicity in Group 1 (OR 3.0 [1.4–6.4], p = 0.003) and had results in a similar direction that did not reach significance for Group 2 (OR 2.1 [0.7–6.2], p = 0.18).

## Discussion

Our study identifies *S*. *mansoni* infection as a novel risk factor for hepatotoxicity among HIV-infected patients on antiretroviral therapy living in regions in which this parasitic infection is endemic. *S*. *mansoni* infection was associated with a three-fold increased odds of hepatotoxicity among HIV-infected adults even after adjusting for other known risk factors for hepatotoxicity. The robustness of our data is attested by the concordance of our findings with past literature; hepatitis B, hepatitis C, and anti-tuberculosis medication use were strongly associated with hepatotoxicity [1]. It is also supported by the ultrasound findings demonstrating the expected increased portal vein diameter, and not only elevated transaminases, in those with *S*. *mansoni* infection. The high rates of hepatotoxicity that we identified in this study, which are notably higher than those reported from other regions of Tanzania, may be due to higher rates of schistosomiasis in this population [16].

This is the first study to our knowledge to show an association between hepatotoxicity and *S*. *mansoni* infection in HIV-infected adults using ART, and one of few to associate hepatocellular dysfunction with this parasitic infection. Two Brazilian studies also showed increased levels of AST, ALT, gammaglutamyltransferase (γ-GT), alkaline phosphatase and total bilirubin in those with *S*. *mansoni* infections [12–13]. Taken together with these Brazilian studies, our work draws attention to the important consequences of *S*. *mansoni* infection not only on portal pressure but also on hepatocellular function. Additional work to explore this impact, particularly in HIV-infected patients, is urgently needed. Only two other studies were conducted in co-infected patients not on ART: one found no increased impact of co-infection on the liver [17], while the other found an association between the intensity of *S*. *mansoni* infection and liver and spleen size but did not observe increased periportal fibrosis in co-infected patients [18]. We are now following up the patients who participated in this study to determine whether treatment of schistosomiasis leads to improvement of hepatotoxicity in this at-risk population.

In our study, hepatitis B surface antigen positivity was significantly associated with hepatotoxicity, as previously documented [1]. Hepatitis B-associated hepatotoxicity has also been shown to be more severe in the setting of schistosome co-infection [19–20]. A recent review concluded that subjects with schistosome and hepatitis B co-infections have a prolonged carriage state, resulting more often in chronic hepatitis with severe cirrhosis and higher mortality compared to patients with hepatitis B infection alone [20]. Our finding of hepatocellular toxicity in *S*. *mansoni*-infected patients affirms this idea, with the more severe cirrhosis being fuelled by the hepatotoxic effects of both diseases in concert.

A limitation of our study is that, because of the use of the CCA test, we were unable to differentiate between schistosome species. Although multiple studies demonstrate that *S*. *mansoni* is by far the prevalent schistosome species in our CTC clinic population, it is possible that several cases of *S*. *haematobium* could have existed in this population and have been included in the analysis. In addition, because our study was cross-sectional and we could not determine how long people had been infected with *S*. *mansoni*, we cannot conclude on a causality link nor do we know the impact of length of co-infection.

In conclusion, our study furthers the importance of prior recommendations that patients starting ART in schistosome-endemic areas should be screened and treated for schistosomiasis [8]. We further urge follow-up liver function testing 3–6 months after ART initiation, particularly in those with schistosomiasis, hepatitis, or anti-tuberculosis treatment. We postulate that treatment of schistosome infections may prevent hepatotoxicity in patients, both during the crucial window of initiating and stabilizing ART regimens and much later as well.

## Supporting information

S1 Checklist STROBE checklist. (DOC)
